# Relationships between physical activity, fundamental motor skills, and body mass index in preschool children

**DOI:** 10.3389/fpubh.2023.1094168

**Published:** 2023-04-12

**Authors:** Fei-Fei Ma, Dong-Mei Luo

**Affiliations:** ^1^School of Sport Science, Beijing Sport University, Beijing, China; ^2^School of Physical Education, Shanxi University, Shanxi, China

**Keywords:** preschool children, physical activity, fundamental motor skill, body mass index, relationships

## Abstract

**Purpose:**

To investigate whether there is a potential relationship between physical activity (PA), fundamental motor skills (FMS), and Body Mass Index (BMI) in preschool children and to further explore the differences in PA and FMS between normal-weight and overweight/obese preschool children.

**Method:**

Participants were 366 preschool children aged 3 to 6 years; 358 completed all tests (194 boys and 164 girls). PA, FMS, body weight, and height were measured by triaxial accelerometer, the Test of Gross Motor Development, Second Edition (TGMD-2), and anthropometry measurement, respectively.

**Result:**

The overall rate of overweight/obesity was 17.0%, with no significant gender difference (*χ*^2^ = 0.628, *p* = 0.428). Older children perform significantly better in both object control skills and locomotor skills. Independent samples t-tests were used to examine the difference between genders on FMS and PA: girls in the 5-year-old group display better locomotor skills (*p* = 0.012) than boys. Boys spent more time on moderate-to-vigorous physical activity (MVPA) and less time on sedentary activity when compared to girls. Results of partial correlation analysis showed that PA was significantly and positively correlated with both locomotor skills and object control skills in preschool children (*p* < 0.01), while there was no correlation between FMS, PA level, and BMI. Results of ANCOVA revealed no significant difference in FMS between normal-weight and overweight or obese preschool children; normal-weight girls had significantly longer MPA and MVPA duration and significantly shorter sedentary periods than overweight or obese girls (*p* < 0.05).

**Conclusion:**

Overweight/obesity in preschool children requires effective measures. PA is positively associated with FMS, while BMI is not potentially related to FMS or PA in preschool years. Overweight or obese girls should develop healthier weight status by increasing MVPA hours and reducing sedentary time.

## Introduction

Overweight/obesity has become a global “epidemic” spreading in developed and developing countries (1). In particular, being overweight and obese in childhood is a global problem that cannot be ignored. By 2014, there were more than 41 million overweight children under five years worldwide. WHO estimates that the number of overweight and obese infants and children will exceed 70 million by 2025 (2). *Nutrition and Chronic Diseases in China Report (2020)* showed that the prevalence of overweight (including obesity) among children under 6 years of age in China is 10% (3), which has drawn significant attention from national authorities (4). Obesity can lead to abnormal growth and development, low immunity, breathing difficulties, psychological problems, social difficulties, etc., and a significantly increased risk of cardiovascular disease, metabolic abnormalities, and metabolic syndrome in adulthood (5). Moreover, it should be noted that overweight and obesity can have adverse effects on the postural status of children (6, 7). Physical inactivity is considered to be the leading cause of childhood obesity (8). Numerous studies have pointed out that adequate physical activity (PA) can reduce the risk of being overweight and obese in young children (9–11). *Physical activity guideline for Chinese preschoolers aged 3–6 years*, which was released in 2022, clearly states the recommended time of preschool children’s daily PA is at least 180 min, at least 60 min of which should be moderate-to-vigorous physical activity (MVPA), and sedentary behavior should be minimized (12).

Fundamental motor skills (FMS) are the proficiency of common basic motor skills with specific patterns (13), which can be classified as locomotor skills, object control skills, and stability skills (14). According to Seefeldt, a motor skill development expert, only when children master FMS can they break the critical “motor proficiency barrier” to actively participate in various sports and increase their self-confidence (15). At the same time, a higher FMS proficiency is beneficial for improving health-related fitness and increasing muscle strength and endurance (16). Furthermore, David et al. identified the development of motor skills as a major potential mechanism to promote PA participation in children (13). According to the mountain of motor development theory, children’s FMS are developed and matured at 1 to 7 years and utilized at 7 to 12 years. Therefore, the development of FMS is a key component of early education from birth (17). Therefore, understanding the relationship between FMS and PA levels during early childhood is a critical factor in promoting healthy behaviors.

FMS proficiency in children and adolescents may also be related to their weight status (18–20). Most of these studies reveal overweight/obesity as a constraint on developing FMS (18, 19). Still, some studies have found this correlation only among girls (20), while others show no correlation between motor development proficiency and BMI at all ages (21). Results and conclusions among studies are inconsistent. Thus further research is needed, particularly in the preschooler group, which is less studied.

Therefore, it is necessary to understand the relationship between BMI, PA, and FMS for further development of effective interventions for preschool children with different weight statuses. Furthermore, this study discusses the difference in PA and FMS levels between preschoolers with different weight statuses. This study serves as a comparison to existing research and, to the best of our knowledge, has not been previously reported among Chinese preschool children.

## Study methods

### Participants

This study recruited 366 physically and mentally healthy children from three kindergartens in Beijing and obtained informed consent from their parents/guardians before the study began. After excluding missing and invalid data, 358 children entered the follow-up analysis (194 boys and 164 girls). All children underwent anthropometry measurement, FMS, and PA tests. Ethical approval was obtained from the ethics committee of the School of Sport Sciences, Beijing Sport University.

### Measurements

Standing height was measured with a stadiometer (TZG, Shanghai). The stadiometer was calibrated before the test began. Have the child stand erect on the floorboard of the stadiometer with his or her back to the vertical backboard. The child’s buttocks, scapulae, and head are in contact with the vertical backboard. The child’s head is maintained in the Frankfort Horizontal Plane position. After the measurement is read, it should be recorded to the nearest 0.1 cm. Weight was measured with an electronic digital scale (HBF-255 T-W). Have the child stand on the center of the weight scale platform. Record the weight to the nearest 0.1 kg. Body Mass Index was calculated by formulation BMI=Weight/Height^2^ (kg/m^2^). The child’s weight status was classified as normal, overweight, and obese by BMI (22).

### Fundamental motor skills

Children’s FMS were evaluated using the Test of Gross Motor Development-2 (TGMD-2). The TGMD-2 evaluates children’s 6 locomotor skills and 6 object control skills. Locomotor skills include run, hop, gallop, leap, horizontal jump, and slide. Object control skills include striking a stationary ball (from a tee), stationary dribble, catch, kick, overhand throw, and underhand roll. Each skill was broken down into 3–5 components, and each component was scored as either a 1 or a 0. Examiners verbally guided and demonstrated each skill; then the child performed that skill twice. Examiners recorded and rated children’s performance, and sum raw scores for six locomotor skills (0–48) and sum raw scores for six object control skills (0–48) were calculated. Each examiner was trained and supervised by an experienced instructor. The intraclass correlation coefficient (ICC) was 0.94–0.98 on locomotor skills and 0.91–0.95 on object control skills.

### Physical activity

Children’s PA levels were assessed using triaxial accelerometers (ActiGraph GT3X+) for 7 consecutive days. Parents and teachers were given verbal and written instructions on the proper use of the accelerometer and were asked to record children’s activities. The accelerometer was worn on the child’s right hip at the anterior superior iliac spine (ASIS), and the child was only allowed to remove it when showering or sleeping. The sampling frequency was 100 Hz, and activity counts were analyzed with 15 s/epoch (23). Only days worn time more than 600 min were considered valid, and children wore them for a mean of 10.7 ± 1.5 h. Zero activity periods of 20 min were interpreted as non-wear time, and counts <0/min or ≥ 15,000/min were excluded because these values are not considered biologically plausible (24). The valid days included in the analysis were 2 weekdays and 1 weekend. The cut-off point for vector magnitude proposed by Butte et al. was used to calculate the duration of low (820 < counts/15 s ≤ 3,907), moderate (3,907 < counts/15 s ≤ 6,111), and vigorous (counts/15 s > 6,111) intensity physical activity (25). Equipment initialization and data analysis were conducted by ActiLife software (version 6.13.3).

### Statistical analysis

The data were analyzed in SPSS25.0 software. Descriptive statistics and frequency calculations were performed for demographic, anthropometry, PA, and FMS variables. A Chi-square test was used for comparative analysis of overweight/obesity rates between genders; an Independent Samples T-test was used for comparative analysis of PA levels and FMS levels between male and female preschoolers of the same age group; ANOVA and *post hoc* analysis were used to examine the differences in PA and FMS levels among ages in both genders; and a partial correlation analysis was used to investigate the correlation between BMI, PA, and FMS levels. Analysis of covariance (ANCOVA) was used to compare the BMI, PA, and FMS levels between normal and overweight/obese subjects, with a significance level of *p* < 0.05.

## Results

Three hundred and fifty-eight preschool children aged 3–6 years (183 boys and 175 girls) from Beijing participated in the study. The overall overweight/obesity rate is 17.0% [61/358, 95%CI (13.1–21.0%)], and the rate of boys was 18.6% [34/183, 95%CI (12.9–24.3%)] and 15.4% [27/175, 95%CI (10.0–20.8%)] for girls, with no significant gender difference identified (*χ*^2^ = 0.628, *p* = 0.428). The mean age of children was 4.48 years, and the mean height, weight, and BMI were 110.3 cm, 19.1 kg, and 15.7 kg/cm^2^, respectively, as shown in Table 1.

**Table 1:** Subject characteristics.

Age group (year)	3	4	5	6	Total
Gender(M:F)	47:44	47:43	45:47	44:41	183:175
Overweight/obese(M:F)	8:6	10:7	7:7	9:7	34:27
Height(cm), *M*(SD)	101.3(4.9)	107.6(5.0)	114.0(5.7)	118.7(5.5)	110.3(7.7)
Weight(kg), *M*(SD)	16.2(2.6)	18.2(2.8)	20.5(4.9)	21.8(4.6)	19.1(4.1)
BMI(kg/cm^2^), *M*(SD)	15.8(1.6)	15.7(1.7)	15.7(2.7)	15.4(2.2)	15.7(2.1)

**Figure 1:**
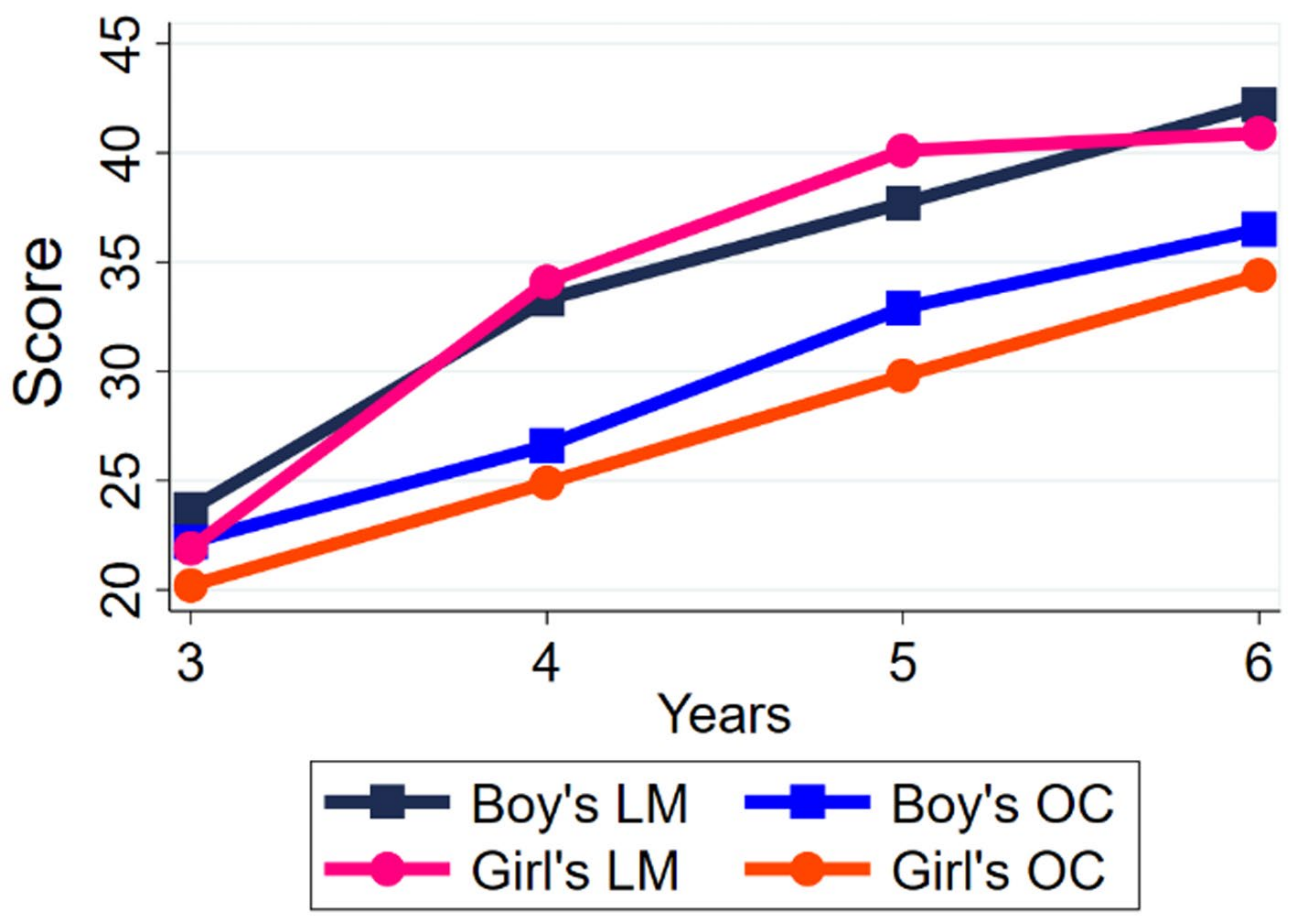
FMS scores (LM = locomotor skills; OC = object control skills).

The ANOVA and *post hoc* analysis were used to compare the FMS and PA levels between ages. The results revealed significant differences in locomotor and object control skills between ages (see Table 2). The significant levels of the 5 and 6 years groups were *p* < 0.05, and the rest age groups were *p* < 0.01. Figure 1 shows the levels of locomotor skills and object control skills between ages. In terms of PA levels, the VPA levels were significantly higher in the 5 (*p* = 0.022) and 6-year-old (*p* = 0.001) groups than in the 3-year-old group.

**Table 2:** Participants’ FMS, SE, and PA.

Ages(year)	3	4	5	6
LM, *M*(SD)	23(6.6)	33.7(6.0)	38.9(5.0)*	41.6(4.3)
OC, *M*(SD)	21.4(6.1)	25.8(5.1)	31.3(5.0)*	35.5(4.8)
SE, *M*(SD)	434.7(40.0)	437.7(40.0)	430.7(29.4)*	431.7(35.7)*
LPA, *M*(SD)	41.2(11.5)	42.0(8.7)	41.3(9.0)*	40.6(10.5)*
MPA, *M*(SD)	31.8(10.8)	33.2(9.5)	32.4(10.9)	32.4(11.4)*
VPA, *M*(SD)	14.2(8.4)*	16.1(7.1)*	17.4(8.3)	17.7(8.3)*
MVPA, *M*(SD)	46.0(17.3)*	49.3(15.5)*	49.8(17.8)	50.1(18.4)*

LM = locomotor skills score; OC = object control skills score; SE = sedentary time; LPA = low intensity PA; MPA = moderate PA; VPA = vigorous PA; MVPA = moderate and vigorous PA. *Represent significant differences between genders (*p* < 0.05) in the same age group.

**Figure 2:**
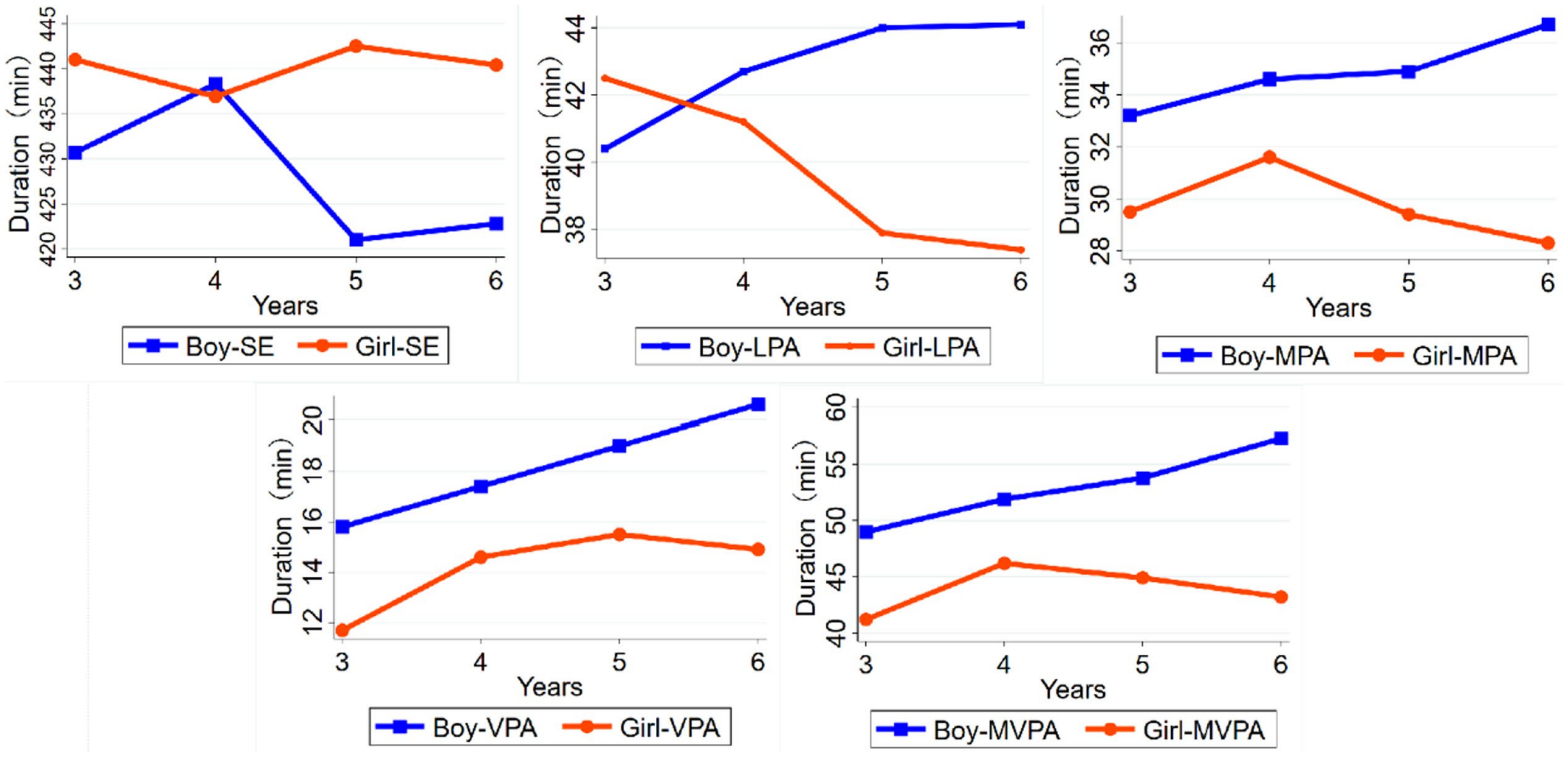
SE and PA levels.

The independent samples t-test was used to compare the FMS and PA levels between the same age group: the 5-year-old girls had better locomotor skills (*p* = 0.012) than boys, but the 5-year-old boys had better object control skills (*p* = 0.001) than girls (Figure 1). In addition, the MPA, VPA, and MVPA levels were significantly higher in the 6-year-old boys (*p* < 0.05) than in girls (Figure 2).

Table 3 displays the correlations between FMS, PA, and BMI in young children. The partial correlation analysis method was used to exclude the interference of age and gender. The results showed that all parameters of PA were significantly and positively correlated with locomotor and object control skills (*p* < 0.01); locomotor and object control skills were not correlated with BMI.

**Table 3:** Correlation between participants’ PA, FMS, and BMI.

	SE	LPA	MPA	VPA	MVPA
LM	0.088	0.143**	0.207**	0.280**	0.259**
OC	−0.008	0.121*	0.138**	0.164**	0.162**
BMI	−0.058	0.097	0.089	0.048	0.093

*Represents *p* < 0.05, **represents *p* < 0.01.

When FMS and PA were compared between normal and overweight obese preschoolers using ANCOVA, it was found that there were no significant differences in FMS levels between normal and overweight obese preschoolers; normal-weight girls had more MPA and MVPA time and less sedentary time compared to overweight/obese preschoolers (*p* < 0.05; Table 4).

**Table 4:** Normal-weight vs. overweight/obese children’s FMS and PA.

	Variables	Normal-weight vs. overweight/obese (boys)		Normal-weight vs. overweight/obese (girls)	
		*F* value	*p* value	*F* value	*p* value
FMS	LM	0.055	0.814	1.326	0.251
	OC	0.853	0.357	0.21	0.648
	FMS	0.483	0.488	0.342	0.56
PA	SE	0.035	0.852	5.284	0.023*
	LPA	3.674	0.057	1.395	0.239
	MPA	1.723	0.191	5.202	0.024*
	VPA	0.443	0.506	3.196	0.076
	MVPA	1.258	0.263	5.116	0.025*

*Represents *p* < 0.05.

## Discussion

This study investigated the interrelationships between weight status, PA level, and FMS proficiency in young children. Three hundred and fifty-eight preschool children completed this study, with an overall overweight obesity rate of 17%, similar to the study by Wang Jing et al. on young children in Beijing (26), with no gender differences in overweight/obesity rates.

### Age differences and gender differences in PA and FMS

The overall trend of increasing duration of moderate-to-vigorous physical activity with age in this study may be because older children usually have higher cognitive levels and more mature physical development. Therefore, age is an important factor in young children’s participation in moderate-to-vigorous physical activity. Pate et al. found that boys participated in PA longer in total and moderate-to-vigorous intensity times than girls (27). The findings of other studies also support that boys are more active and involved in more moderate-to-vigorous physical activity than girls during early childhood (28, 29), similar to the present study results. This may be because boys are usually more active and better at all sports, giving them more opportunities to participate in sports longer. While girls are more involved in creative play and activities, such as drawing and painting, crafts, etc., thus increasing their LPA and sedentary time.

In terms of motor development levels, there were significant differences in motor development between all age groups and an overall linear trend of increase. Figure 1 shows that boys generally had better object control skills than girls, which is consistent with most studies (8, 30). This is because boys are more likely to engage in ball sports, which increases their mastery of object control skills. Bandeira reported that boys performed better in throwing and hitting skills (31), while Pablo’s results concluded that boys had a greater mastery of hitting and dribbling skills (32). Locomotor skills did not show regular gender differences, with girls outperforming only boys in the 5-year-old group. However, most studies in recent years have reported no significant differences in displacement skills between boys and girls (33–35), and some studies have reported that boys scored higher than girls in both locomotor and object control skills (36, 37). Support for the idea that girls’ locomotor skills are superior to boys’ may be due to the fact that girls develop earlier than boys, resulting in differences in physical capacity. As for the possibility of no gender differences in this study, it may be because in the preschool stage, children have similar physical development without gender advantages.

In addition, Figure 1 shows that the development of locomotor skills is faster than that of object control skills, which is mainly due to the fact that 3–6 years old is a crucial period for the development of large muscle motor skills, and compared to displacement skills, object control skills require more hand-eye coordination and control while examining the power of large muscles, which requires more attention, analysis, and use of external information (38). In addition, the test instrument selection is also crucial, as the TGMD-2 emphasizes the degree of movement completion, and each indicator corresponds precisely to each part of the limbs and trunk. However, due to cultural differences, Chinese children are less exposed to baseball-like movements, such as hitting a stationary ball with both hands and throwing a ball overhand in the object control skills category, which makes it more difficult to develop a mature movement pattern.

### Correlation of PA, FMS, and BMI in preschool children

The results of this study showed that PA was significantly and positively correlated with both locomotor skills and object control skills. Studies have shown that FMS are closely related to PA, and FMS are a necessary foundation for physical activity. The development of physical activity can promote the improvement of fundamental movement skill ability (39, 40). Since this study was cross-sectional, it is difficult to determine whether higher levels of PA contributed to higher levels of FMS or higher levels of FMS contributed to higher levels of PA. However, in a longitudinal study of school-age children, Elizabeth et al., concluded that to increase PA engagement levels, FMS mastery is needed, rather than increasing the time spent on PA to increase FMS mastery (41). Similarly, Crane and his colleagues have identified the development of FMS in early childhood as a key behavioral determinant of PA habits and noted that developing FMS early in life provides a stimulus for developing lifelong PA (42). Thus, understanding the relationship between FMS and PA in early childhood can help us further research to improve lifelong PA. In addition, most studies similarly consider proficiency in FMS as a prerequisite for improving PA, as learning locomotor skills is necessary for participation in PA (43).

Regular physical activity (activity that consumes energy through skeletal muscle contraction) is an effective means of promoting children’s physical and mental health development, and can effectively reduce the incidence of obesity and a series of diseases caused by obesity (44). Studies have concluded that overweight/obese children have lower levels of PA and longer sedentary time compared to their normal-weight peers (45, 46). Still, no significant relationship between BMI and PA was found in this study (as shown in Table 3). In further gender comparison of normal and overweight obese children, no significant difference in PA levels was found between normal-weight and overweight/obese boys. Still, normal-weight girls spent significantly more time on vigorous physical activity and less time on sedentary time. The gender differences may be due to the influence of socialization factors, where children participate in specific types of activities by gender, including free play and structured activities (47–49). In addition, studies have confirmed that girls have higher self-esteem in early childhood than boys (45–50). For overweight and obese girls, although motor development does not differ significantly from that of girls of the same age, demonstrated ineptitude or “clumsiness” in movement tends to affect their self-esteem. It leads them to stop or abandon PA, thus choosing activities with lower levels of PA or longer sedentary time. Therefore, it was found that preschool girls’ PA levels were more likely to be influenced by their weight status.

This study found no statistically significant association between the level of FMS development and BMI in young children. Previous studies have had inconsistent findings regarding the relationship between preschoolers’ weight status and their FMS development level. It is generally assumed that children with a high BMI have lower levels of FMS because being overweight typically has a more significant influence on running, jumping, and items that require dynamic body coordination (51–53). In addition, a higher level of FMS in children can improve their ability to participate in other PA, and the improvement of PA ability can help maintain a healthy weight. However, in a study of young Chinese children using the TGMD-2 test, FMS were not correlated with BMI (54), and other studies have supported the idea that the two are unrelated (21, 55). It may be that preschool children have not fully learned and mastered these fundamental movement skills (56), thus not effectively improving their PA level, which results in no significant relationship with BMI.

Stodden et al. proposed a theoretical model of the relationship between PA, FMS, perceptual-motor skills, health-related physical fitness, and obesity in children (13). It is emphasized that PA is an important moderator of FMS and obesity, but the effects of other moderating variables are equally important. This study did not find a relationship between FMS and BMI, this may be because the influence between these factors is a dynamic interaction process over a long period. The young age of preschool children, who were the subject of this study, maybe the reason for the absence of a significant relationship. The development of FMS is sustainable (57), and there is growing evidence of a moderate-to-strong positive correlation between enhanced levels of FMS with age and physical fitness, particularly in adolescence (58, 59).

Although this study explored the relationship between BMI, FMS, and PA in preschool children, there are several limitations that need to be addressed. Firstly, due to the cross-sectional design of this study, it was not possible to examine the reciprocal effects among the three factors. Future longitudinal studies are needed to gain a better understanding of the interplay among these factors. Additionally, the use of BMI to classify weight status has limitations as it cannot distinguish between body fat and lean body mass (60). Therefore, for further research on children, body fat measurement (such as using a skinfold caliper) should be used instead of BMI to measure weight status.

## Conclusion

High overweight/obesity rate in preschool children require effective measures to be implemented by school, family, and society. Boys had higher PA levels than girls. There was no correlation between BMI, FMS, and PA. Given the importance of BMI, FMS, and PA for promoting healthy growth and development in children, it is crucial to track and explore the changing trends and mutual promotion effects of these factors with age. For overweight/obese girls’ healthy development, time spent on MVPA should be increased and sedentary time should be reduced.

## Data availability statement

The original contributions presented in the study are included in the article, further inquiries can be directed to the corresponding author.

## Ethics statement

The studies involving human participants were reviewed and approved by ethics committee of the School of Sport Sciences, Beijing Sport University. Written informed consent to participate in this study was provided by the participants’ legal guardian/next of kin.

## Author contributions

F-FM contributed to conceptualization, software, data curation, and writing (original draft preparation), investigation, and writing (reviewing and editing). D-ML contributed to methodology and visualization. All authors contributed to the article and approved the submitted version.

## Funding

This project was supported by the National Social Science Fund, Project name: Development and application of evaluation method of preschool children’s exercise intensity and artificial intelligence evaluation system [BLA200222].

## Conflict of interest

The authors declare that the research was conducted in the absence of any commercial or financial relationships that could be construed as a potential conflict of interest.

## Publisher’s note

All claims expressed in this article are solely those of the authors and do not necessarily represent those of their affiliated organizations, or those of the publisher, the editors and the reviewers. Any product that may be evaluated in this article, or claim that may be made by its manufacturer, is not guaranteed or endorsed by the publisher.
